# Transforming Connections: A Trauma-Informed and Attachment-Based Program to Promote Sensitive Parenting of Trans and Gender Non-conforming Youth

**DOI:** 10.3389/fpsyg.2021.643823

**Published:** 2021-07-26

**Authors:** Antonia Dangaltcheva, Chris Booth, Marlene M. Moretti

**Affiliations:** ^1^Psychology Department, Simon Fraser University, Burnaby, BC, Canada; ^2^Maples Adolescent Treatment Centre, Vancouver, BC, Canada

**Keywords:** adolescence, parenting, attachment, gender, intervention

## Abstract

Gender non-conforming and trans youth experience high rates of bullying and victimization, placing them at risk for serious mental health challenges. Parent support is one of the most significant protective factors in this population, and yet few programs are specifically developed to promote parenting sensitivity, understanding, and acceptance. *Connect*, a trauma-informed and attachment-based group program for caregivers of at-risk adolescents, has been shown to reduce parent stress and depressed mood, increase parents' sense of efficacy and satisfaction, and reduce parent-teen conflict. Teens benefit from increased attachment security and improved mental health and well-being. Treatment effects have been documented to continue for up to 2 years post-treatment. This paper describes the adaptation of the *Connect* program to create a new program, *Transforming Connections*, for caregivers of transgender and gender non-conforming youth. Participants in the first three groups were 20 parents of 16 gender non-conforming youth (ages 12–18). Common themes in group discussions related to gender included: coming out, connecting with peers, affirming pronouns/names, medical transition, parental reactions (e.g., confusion, isolation, grief, acceptance), and concerns about safety and mental health. All parents completed the full program, attending on average 9 of 10 sessions. Caregivers reported feeling respected, safe, and welcomed in the program and indicated that learning about attachment enhanced their understanding of their teen and their gender journey as well as themselves as a parent. Additionally, all parents reported applying the ideas discussed in the group frequently (60%) or somewhat frequently (40%). The majority indicated that their relationship with their teen had improved somewhat (65%) or a great deal (20%). Findings provide positive preliminary evidence of the fit and value of *Transforming Connections* for these families.

## Introduction

Transgender and gender non-conforming youth are at risk for significant family conflict and rejection, peer bullying, and gender-based violence and victimization (Grossman and D'Augelli, 2007; Hendricks and Testa, 2012; Reisner et al., 2014). These adverse experiences undermine mental health and well-being and increase vulnerability for depression, self-harm, and suicidality (Clark et al., 2014; Reisner et al., 2015; Holt et al., 2016). For example, a cross-Canada survey of 923 trans youth between ages 14 and 25 found that nearly two thirds of youth reported engaging in self-harm compared to only 17% of same aged gender conforming peers; approximately one third had attempted suicide in the last year compared to 6.5% of peers (Veale et al., 2015, 2017). Five years later, an updated version of the cross-Canada survey consisting of 1519 youth between the ages 15 and 24 continued to describe alarmingly high levels of self-harm and suicidal ideation, but youth with supportive families were less likely to report suicidal thoughts and emotional distress (Taylor et al., 2020).

Caregiver support is a critical protective factor that increases resilience, promotes mental health, and mitigates risk of suicidality among gender non-conforming youth (Katz-Wise et al., 2018; Grossman et al., 2019). Simons and colleagues (Simons et al., 2013) found that higher levels of parental support among transgender youth (ages 12–24) was associated with fewer depressive symptoms and higher life satisfaction. Similarly, Ryan and colleagues (Ryan et al., 2010) reported that greater family acceptance among LGBT young adults (ages 21-25) was linked to higher levels of self-esteem, greater life satisfaction, and fewer mental health problems. Conversely, suicidal risk was eight to nine times higher among those who reported family rejection (Ryan et al., 2010).

Low levels of parental support are common among transgender youth. Travers et al. (2012) found that 42% of transgender youth (ages 16–24) reported their parents were “not very” or “not at all” supportive; while another 25% reported their parents were only “somewhat supportive.” Only 34% reported their parents were “very supportive.” Youth who described their parents as “very supportive” were far more likely than those with lower levels of parental support to report very good or excellent mental health (70 vs. 15%), higher self-esteem (64 vs. 13%), and life satisfaction (72 vs. 33%). They were also far less likely to report depression (23 vs. 75%) and to attempt suicide (4 vs. 57%).

Even though there has been some debate in the literature as to the relative protective benefits associated with parental support for youth with gender dysphoria (Schumm and Crawford, 2020), the larger literature consistently underscores the importance of parental support and secure attachment in promoting adolescent well-being. There are likely diverse trajectories among youth with gender dysphoria as they strive toward self-understanding, self-expression, and developing connections in the community. Undoubtedly, a secure relationship with caregivers that encourages open communication, facilitates a supported process of identity exploration in adolescence, which can be helpful to all youth.

To date, health promotion strategies have focused primarily on supporting trans and gender non-conforming youth through changing public policy in health, school, and social domains to reduce social stigma and victimization, as well as providing gender affirming interventions directly to youth that support their gender journey and self-esteem. Such efforts are critical in promoting social inclusion, safety, and youth well-being. However, less attention has focused on developing interventions to support parents and caregivers of gender non-conforming youth with the goal of promoting gender affirming and sensitive caregiving. Indeed, parents who struggle to understand their teens' gender journey are sometimes viewed in critical and condemning ways, which does little to promote their acceptance and development of sensitive caregiving skills. Evidence-based interventions are needed to provide a supportive context for parents to explore and accept complex emotions and the very real challenges that their teens may face (Ryan et al., 2010; Gray et al., 2012; Simons et al., 2013; Veale et al., 2015; Andrzejewski et al., 2021).

Preliminary evidence suggests that support groups for parents of trans youth ranging from 3 to 21 years may be beneficial to caregivers (Menvielle and Tuerk, 2002; Rosenberg, 2002; Di Ceglie and Thümmel, 2006; Menvielle and Rodnan, 2011; Hillier and Torg, 2019; Lawlis et al., 2020). These groups offer a cost-effective method of delivering service to families while reducing feelings of isolation and increasing a sense of connection with other parents who are facing similar challenges. Group discussions generally center around similar themes including identification of the child's gender variance, reactions to coming out, perceived loss and grieving, transitioning, challenging situations, concerns about safety, and uncertainty about the future (Menvielle and Hill, 2010; Riley et al., 2013). To date, such groups have adopted a parent-support group format which may benefit some parents, but the groups are limited in their use of specific treatment components that promote sensitive and responsive parenting skills. Attachment-based treatment models offer a strong foundation to create interventions that are tailored to the unique challenges of caregivers of trans and gender non-conforming youth, to promote parental understanding, sensitivity, and responsiveness, and to retain a supportive and non-judgemental treatment context. Importantly, such approaches are well-suited to understanding and addressing the frequency of insecure attachment and exposure to trauma that has been documented among trans and gender non-conforming youth and their families (Giovanardi et al., 2018; Kozlowska et al., 2021). For example, Kozlowska et al. (2021) found that the frequency of insecure attachment strategies was high among youth with gender dysphoria, especially among those who experienced traumatic loss. Similarly, Giovanardi et al. (2018) showed that 73% of adult with gender dysphoric were insecurely attached with their parents and over half reported four or more early experiences of trauma.

Guided by research demonstrating the efficacy of attachment-based interventions for children, the *Connect* program is a trauma-informed intervention that collaboratively engages caregivers and builds sensitive parenting skills that promote attachment security in teens (Moretti et al., 2017; Moretti, 2020; http://connectattachmentprograms.org/). Designed for delivery by a wide-range of mental health and education professionals, this ten-session program (1.5 h weekly) is delivered by two trained facilitators who guide groups of 8–14 parents through emotion-focused, experiential, and reflective exercises that increase their understanding of trauma, attachment, and adolescent mental health. Sessions begin with a didactic introduction of an attachment principle and include experiential activities that help caregivers develop skills to identify, understand, and respond to the needs of their teens. The group content focuses on the building blocks of attachment: caregiver sensitivity, shared partnership and mutuality, reflective functioning, and effective dyadic affect regulation. Parents learn to “step back” in their interactions and to “step into” their teen's shoes. They practice recognizing their own thoughts and feelings in these situations and temporarily setting them aside. They focus on reframing their adolescent's behavior using an attachment framework, modulating their own emotional responses, and mindfully utilizing strategies to support their relationship, while clearly setting limits and expectations. The experiential components of the model can be tailored to unique cultural diversities and specific community concerns can be integrated.

The effectiveness of *Connect* has been demonstrated in a series of randomized and quasi-experimental clinical trials. Studies have demonstrated significant and lasting reductions in teens' symptoms of depression, anxiety, and conduct problems in clinical populations of teens with serious behavior problems and other mental health conditions (Moretti and Obsuth, 2009; Moretti et al., 2015). Parents also benefit from *Connect*, shifting to a more positive view of themselves as a parent and a more positive view of their teen (Moretti et al., 2012). Their relationship with their teen moves toward a greater sense of partnership and mutuality, increased trust, autonomy and attachment security, and they report significant and lasting decreases in depressed mood and caregiving stress, as well as increases in parental sense of competence (Moretti, 2019).

Evidence supporting the efficacy of *Connect* has been well-documented in independent clinical trials, including a randomized clinical trial of 908 parents in Sweden enrolled in parenting programs for child behavior problems in which outcomes were sustained over a two-year follow up period (Stattin et al., 2015; Högström et al., 2017). Moreover, *Connect* was the only parent program in this study to produce significant additional improvements from post-treatment through follow-up. Similar findings were reported in a quasi-experimental study in Italy (Giannotta et al., 2013; Ozturk et al., 2019) and in a randomized clinical trial of Somali-born refugee parents living in Sweden (Osman et al., 2017). More recently, these findings have been replicated in a population of over 900 caregivers and youth in Canada, demonstrating significant improvements in a number of treatment outcomes as early as mid-treatment (5 sessions) that were sustained for up to 18 months. These effects were evident on both parent and youth report measures (Moretti, 2019).

Although promising, the fit and perceived usefulness of *Connect* has yet to be established with gender non-conforming youth and their caregivers. The clear clinical structure of the *Connect* program is flexible to adaptations that ensure the unique challenges and needs of specific caregiver populations can be well-represented and integrated throughout experiential learning exercises. The current study reports on the adaptation of the *Connect* program to address the needs of parents of gender non-conforming and trans youth and measure the effectiveness of the program. This newly adapted program was titled *Transforming Connections*, acknowledging the inherent journey, which is necessary for both caregivers and youth, individually and in their relationship.

## Methods

### Participants

Participants were 20 parents (14 mothers, 6 fathers) of 16 gender non-conforming youth (ages 12–18), who participated in one of the pilot groups delivered in this study. Parents ranged between 32 and 59 years old (*M* = 49.05, *SD* = 8.10). The majority self-identified as Caucasian (90%; *n* = 18); 5% as Aboriginal (*n* = 1); and 5% as Asian (*n* = 1). In terms of formal education, all parents reported having their high school diploma and most (75%; *n* = 15) had post-secondary degrees. In describing their challenges, most parents expressed concerns about youth self-harm (63%; *n* = 10). The majority of teens were seen by a counselor in the community (69%; *n* = 11), and some were seen by a psychiatrist (25%; *n* = 4); however, several were receiving no additional mental health support (25%; *n* = 4). A minority of parents (15%; *n* = 3) had sought out therapeutic services for themselves via mental health or other support groups in the community. With respect to their teen's gender transition, the time elapsed since youth first expressed their gender identity to their parents ranged between 2 months to 5 years; the average time was approximately 17 months (*M* = 16.88, *SD* = 16.28). Six youth had begun taking hormones or hormone blockers. In terms of participation, parents attended 9.2 of 10 sessions on average (*SD* = 0.93).

### Program Adaptations and Delivery

*Transforming Connections* was facilitated by doctoral level clinicians with 6 years of cumulative clinical experience working with gender non-conforming youth and their families. All sessions were videotaped and reviewed by the clinicians and their supervisors, including the *Connect* program developer (Moretti) and a consulting psychiatrist who specializes in transgender and gender non-conforming youth (Booth). Weekly supervision sessions focused on how to adapt program exercises to address the unique challenges of parents in the groups and the issues they raised, while ensuring program fidelity, adherence to session structure, exercises, and goals. Members of the Transgender Health Information Program with lived experience and other health professionals in the community also guided the development of various aspects of the program at several key points in the study, including suggestions regarding ways to manage challenges that they had experienced in facilitating a parent support group.

*Transforming Connections* consisted of ten sessions, which progressively built upon each other. During the initial Welcome Session, parents introduced themselves and shared some of their experiences and concerns, as well as their hopes for what they could achieve in the group. Each subsequent session introduced a key principle related to attachment and parenting teens. Sessions included reflection exercises, role-plays performed by the two group leaders, and discussions that focused on helping parents “step back” from their emotional reactions to difficult situations and “step into” their teen's experiences.

To increase the fit of these exercises, the content of each session was adapted to touch on challenges parents experienced with their teen in relation to their gender journey. Role plays showing challenging parent-teen interactions were also adapted. For example, the new scenarios included a child being outed at school, a parent using the child's birth name instead of their gender-affirming name, a child expressing curiosity and excitement for medical transition, and a child opting out of activities (e.g., leaving the soccer team, seeing grandparents) because they do not feel accepted or safe in those environments. After watching the role plays, parents were encouraged to reflect on the thoughts and feelings of the teen in the role play, and their behavior in terms of attachment needs and their gender journey. Parents also reflected on the thoughts and feelings of the parent in the role play and discussed whether their behavior “left the door open” in the relationship or created distance. Parents then discussed how a parent might react differently to strengthen their relationship. At each step, parents were encouraged to think of how this might apply to their teen.

Other changes included addressing the special challenges experienced by parents of trans and gender nonconforming, including disruption of other family relationships, and the need to recognize their own needs and to balance these with those of their teen. Throughout the program, parental reflection and empathy in relation to their teen's gender journey was promoted and examples of how parents might respond to these challenges were discussed and illustrated. Parents often spoke about different aspects of their teen's gender journey, in the context of sharing knowledge, experiences, and reactions with one another. Full details regarding the program adaptations can be requested from the senior author (Dangaltcheva).

Apart from these adaptations that ensured the unique challenges of parents in the groups were addressed, few other program changes were needed as the core attachment principles and the core skills of reflective function, sensitivity, and dyadic affect regulation applied universally. The group also retained a collaborative stance with the parents, avoiding “teaching” them how to parent and instead promoting reflection, autonomy and parenting skills in meeting inevitable challenges.

### Procedure

Ethical approval was obtained from the Simon Fraser University (SFU) Research Ethics Board (REB). All parents provided consent and completed measures prior to and upon completion of *Transforming Connections*. Guidelines for conducting qualitative research in psychology (Elliott et al., 1999) were carefully considered and informed the analytical strategy and procedures were consistent with guidelines for ethical research with transgender populations described by the Canadian Professional Association for Transgender Health (Bauer et al., 2017) and other experts in this field (Adams et al., 2017).

#### Group Themes

Sessions were videotaped and transcribed by the senior author (Dangaltcheva). Transcription focused on parents' reflections related to gender, youth mental health, and the parent-teen relationship. To establish interrater reliability, a research assistant was trained to identify and transcribe relevant aspects of the videotapes following the same coding scheme. Agreement between the two coders was 75% of all utterances that were transcribed.

To identify themes, template analysis was selected because it a widely used and flexible technique in qualitative research in psychology (Brooks et al., 2015). Template analysis allows the author to use apriori themes and hence seemed to be an ideal approach due to the author's previous knowledge of the literature and experience with facilitating the group. An initial template of themes was developed based on previous research and the author's notes from reviewing the videotaped sessions for the purposes of clinical supervision. The author assigned themes to the verbatim interview transcript using NVIVO 11 (QSR International Pty Ltd, 2015). Each utterance was coded separately and could potentially receive one theme, multiple themes, or none. Given the exploratory nature of this research, 20% of the sessions were double coded by a research assistant to establish reliability. Good reliability was achieved (69.3%).

### Measures

#### Treatment Engagement and Client Satisfaction

The last session provided an opportunity for parents to discuss their experience and provide feedback by completing a post-treatment survey and participating in a semi-structured feedback interview. Parents first completed the anonymous survey which included six questions, rated on a 4-point scale ranging from very helpful to unhelpful. Questions focused on assessing the extent to which the program was helpful in understanding their teen and their gender exploration, themselves and their parenting behavior, as well as the degree to which they applied what they learned. The remaining questions asked about whether they felt accepted and supported in the group, and the extent to which the group met their expectations. Finally, open ended questions elicited recommendations for improvements in the program, cultural fit and sensitivity, and challenges in attending.

The interview was facilitated by a different clinician, who was familiar with *Connect* and had experience working with gender non-conforming youth and their families, to ensure caregivers felt comfortable to provide honest feedback even if critical. Specifically, the leader inquired about the caregivers' sense of program fit with their needs, program acceptability, perceived value in relation to their teens' functioning, and their sense of efficacy and satisfaction as a parent.

## Results

### Group Themes

The themes that were identified in the transcripts are listed in Table 1 and described in more detail below.

**Table 1:** Themes across sessions.

**Themes**	**Number of themes across sessions**
Coming out	77
Connecting with peers	52
Names and pronouns	57
Medical transition	38
Grief	83
Concerns	140
Acceptance of gender	104

#### Coming Out

Some parents described feeling shocked when their teens first came out to them, whereas others noted that there had been signs when their children were younger, but they were uncertain of what these signs meant. In addition, parents reported feeling anxious about their teens coming out to others but were happily surprised that when teens came out to peers or came out online, the reactions they received were largely positive. Parents shared that sometimes coming out to their extended family was a difficult process. Some anticipated that their family may not be supportive and delayed telling them; however, it became difficult to consistently keep in mind who their teen had or had not come out to and this in itself was stressful. One parent shared how frustrated they felt trying to prove to the rest of the family that their teen was trans, and this made them realize how difficult it was for their teen who faced the challenge of having to prove their gender identity to the world on a daily basis. Others shared having to cut off relationships with family and friends who were not supportive. All these situations caused a lot of anxiety for teens and parents alike. These comments highlighted the importance of understanding the role of family attachment more broadly in supporting parents of trans and gender non-conforming youth.

#### Connecting With Peers

Parents noted that for their teens the need to be accepted by peers was strong, but unfortunately many of their teens were isolated. Parents acknowledged connecting with peers online may be fulfilling an attachment need and can function as a “lifeline and anchor,” but at the same time, they also referred to it as a “double edge sword.” They came to understand the attachment needs of their teens to feel connected with peers and discussed how to balance this recognition with their anxiety about their teen's safety, especially when their teens wanted to meet their online friends in real life.

#### Names and Pronouns

Parents acknowledged that they encountered difficulties with their teen's pronouns and names changing. They discussed changing their teen's birth certificates and passports and shared their own feelings about the process. Some parents found it particularly challenging to adjust to using the pronouns “they/their/them,” and others admitted that they continued to make mistakes even years later. They shared that they did not intend to be disrespectful but deepened their understanding of how these mistakes impacted their teen and their relationship. Parents also discussed challenges navigating situations in which their teens are misgendered by others. Some spoke about having to advocate for their children while others discussed ways that they helped their teens cope with their feelings after being misgendered.

#### Medical Transition

Parents were keen to discuss hormones and surgery with one another as they could not broach these topics with their friends. They acknowledged that although they are trying to be supportive and respectful of their teens, at times it was difficult to have these discussions because they felt they were “losing control.” When asked whether their concerns were getting in the way of connecting to their teens, one parent stated, “It was totally getting in the way, me not loving the whole process.” They reflected that their reaction had created distance in their relationship and as a result their teen was less likely to initiate conversations in the future. Parents were also concerned regarding the impact of the steps their teen was currently taking on their development, their future, and their ability to have their own children. One parent stated, “My teen wants to make all these lifelong decisions, and I'm putting on the brakes. I don't want to come across as the bad guy but ultimately I'm the one who is really responsible.”

#### Grief

Parents shared that it was difficult to discuss their family history while being sensitive to their teens, and some had to remove family pictures from their homes because of requests from their teens. Parents missed aspects of their previous relationship and felt immense loss. They acknowledged that these strong feelings were a barrier to them connecting with their teens. For example, one parent stated, “I am watching my little girl disappear, rather than watching my little man grow.” Others emphasized that their teen was still the same person.

Parents also expressed feeling grief regarding their teens no longer participating in activities they once enjoyed, because of their transition. They felt that their teens missed out on opportunities other teens were afforded and they conveyed concerns regarding the ambiguous loss that their teens may experience in the future (Simon and Farr, 2020). For example, parents shared that their own teens had left sports teams for various reasons including wanting to play on a team of their affirmed gender or feeling uncomfortable in change rooms. Unfortunately, teens had lost touch with friends as a result. Parents often felt conflicted because they wanted their teens to be involved and to learn about commitment and responsibility, but they did not want to force their teens to do something they did not want to do. One parent spoke of his sadness for his teen because, “lots of doors have closed and really not many have opened.” Another parent noted, “I find myself grieving all the time because they're missing out on something…but their mental health is way more important.”

#### Concerns

Parents shared concerns regarding their teen's safety in the community, particularly when they venture out independently or need to use a public washroom. One parent shared similar concerns regarding safety when their teen made connections with friends from a youth group who were older. They stated that “creating a community with kids he is comfortable with is important,” but they worried about the influence of older peers and potential exposure to substance use.

Parents also shared concerns regarding their teen's low self-esteem, self-harming behaviors, and mental health challenges. Parents in one of the groups frequently returned to a theme of having teens who are especially vulnerable, and some described them as “fragile.” Parents indicated that they had to be “hypersensitive” and “more protective.” They struggled to set limits because they felt they had to “overcompensate.” Toward the end of the group, parents questioned their perception of their teens. For example, one parent asked, “Are we making them more fragile because we give into them more?” while another questioned, “Am I helping him be codependent on me?”

Finally, parents in the groups shared doubts about their teen's gender identity and worried that it might be “a phase.” Some believed that their teens may change their minds in the future. These doubts seemed more pronounced when parents perceived their teens not to be moving forward with their transition in the way they had expected. Parents whose teens were gender fluid or gender nonconforming appeared to struggle even more, especially when their teen's gender expression seemed inconsistent. These are common concerns that come up for parents in support groups (Di Ceglie and Thümmel, 2006; Menvielle and Rodnan, 2011); however, parents in our groups also recognized how their doubts were affecting their teen and their relationship. For example, one parent stated, “My teen believes that I don't think they are really trans, and therefore they are embarrassed or ashamed. They don't like it. They don't want to open up. They make themselves less the way they want to be.”

#### Acceptance of Gender

Parents in the group discussed the process of accepting their child's gender identity and understanding their experience, including various barriers that got in the way of supporting their teens. For example, they spoke about how the language around gender is always changing and it is difficult to keep up. Parents also expressed confusion about the steps their teens were taking in their gender journey. Additionally, many parents felt isolated from their friends as they were dealing with unique issues in parenting. For some parents, it was difficult to empathize because they could not quite understand what their teen was going through as they could not relate their teen's gender journey to their own experience. One parent shared with respect to their teen's transition, “My teen believes that I don't understand, and they don't try to explain or share their feelings.” Another parent recognized that each time they, “ignore an opportunity to connect, the wall gets thicker.”

At times, parents talked about feeling disconnected from their teens. Often, teens did not want to discuss their mental health or their gender journey with their parents. Toward the end of the group, one parent indicated that some of their takeaways were that their teens felt “unheard” and they had learned to “pay attention.” Parents also expressed instances in which they connected with their teens. For example, one parent shared that her teen was surprised that she agreed to go shopping for a binder. She speculated that her teen initially believed she was not accepting of their gender journey. Her actions challenged her teen's beliefs and ultimately their relationship was left in a better place.

### Parent Feedback

On the Treatment Engagement and Client Satisfaction Questionnaire, all caregivers (*N* = 20) reported feeling respected, safe, and welcomed in the group. Similarly, all parents reported that learning about attachment was helpful (30%; *n* = 6) or very helpful (70%; *n* = 14). They also indicated the group was helpful (35%; *n* = 7) or very helpful (65%; *n* = 13) in enhancing their understanding of their teen, and helpful (60%; *n* = 12) or very helpful (40%; *n* = 8) in enhancing their understanding of themselves as parents. Similarly, most parents rated the group as helpful (70%; *n* = 14) or very helpful (20%; *n* = 4) in increasing their understanding of their teen's gender identity and transition, with two parents indicating that it was not helpful in this regard.

Most parents (60%; *n* = 12) reported applying ideas discussed in the group at least somewhat and 40% (*n* = 8) applied them frequently. The majority (65%; *n* = 13) indicated that their relationship with their teen had changed at least somewhat, while 20% (*n* = 4); reported major changes in their relationship. Of note, those who did not apply these concepts as frequently also reported less change in the relationship, *χ*^2^ (1, *N* = 17) = 5.24, *p* < 0.05, Cramer's V = 0.56. All parents anticipated more change in the future. Overall, parents endorsed somewhat (50%: *n* = 10) or greatly (50%; *n* = 10) improved feelings of efficacy while parenting.

Fourteen parents indicated that they had attended either parenting groups or support groups in the past. In comparison to groups parents had attended in the past, *Transforming Connections* was rated as better (64%; *n* = 9) or much better (25%; *n* = 3). Two parents indicated that it was similar to other groups, but none rated it as worse. In addition, most parents (80%) indicated that they enjoyed both the supportive (e.g., meeting with other parents to share advice) and structured (e.g., attachment principles, role-plays, etc.) aspects of the group. Two parents preferred the supportive aspects, and one parent preferred the structure.

Qualitative themes regarding the impact of the intervention were captured in open-ended questions. Parents reported they were better able to empathize with their teen and they felt more confident in parenting. For example, parents stated, “[The group] helped me understand that when my child is upset/angry/appears selfish/inconsiderate, etc. she is really expressing a connection need,” and, “[The group] helped me try to understand the troubles they face as trans teens.” Another parent noted, “I was pretty freaked out about what my child is going through, but I realized that he is actually the same, pretty well-adjusted kid. It was reassuring. If there are issues in the future, I think I can handle them.” When asked about specific examples of how the group was helpful with parenting, most parents listed that stepping back to reflect on their teens' attachment needs was most helpful. Parents also listed that it was helpful to reflect on and understand their child's experience and their feelings, to consider their own needs, and to feel reassured that they could handle issues in the future.

Regarding the role-plays, most parents reported that they found them relevant. Some listed that they wanted to see more defiance or avoidance in the teen. One parent noted that their child was further along in their transition, so some issues discussed were not as relevant. Other suggestions for new role-plays themes included dealing with low self-esteem, managing crisis situations, and substance use.

In terms of limitations, caregivers in one group reported that their teen had complex mental health needs and engaged in self-harm. Many wanted additional support regarding managing crisis situations. In addition, caregivers faced ongoing challenges as their teens began to transition. They asked for more time to discuss these topics and they wanted more information specifically regarding medical transition. This feedback points to the need to ensure that additional supports and consultations around acute medical and psychiatric challenges can be made available to parents to ensure the safety and well-being of their teens.

Some parents felt that the group was not always specifically targeting issues regarding gender and that it addressed “typical teen behavior,” but others indicated that they felt relieved that they were facing universal parenting issues. One parent noted, “As the group progressed, we delved more into topics with a more specific target of trans issues which was helpful.” Another parent wrote, “Our teens have many issues faced by most teens. But it was great having a group of parents that all shared transgender issues. The role-plays were adapted for us which made it more relevant. The discussions that followed allowed us to explore trans issues as a group.” Parents also asked for more time in general and many of the parents wanted ongoing contact with the group members and leaders.

With respect to other changes, several parents indicated that it would be helpful for teens to attend a parallel group that is structured around the same principles. Other suggestions included more sessions for more time to learn about group members and their teens, initiating a Facebook page with helpful information, and uploading handouts online.

## Discussion

Beyond initiatives to promote the recognition and rights of transgender and gender non-conforming youth, and direct support and services for youth as they transition, there is a need to support their families so in turn they can stand with their teens, providing support and acceptance. This research entailed the adaptation of a trauma-informed and attachment-based intervention with established effectiveness to address the needs of parents of trans and gender non-conforming youth. The structure of *Connect* allowed the consultation team to easily adapt the content of the role-plays to address the common and unique challenges experienced by parents attending the program. Although the adapted role-plays had been discussed in advance, as the groups progressed, new role-plays were created to capture issues with which caregivers struggled. During group sessions, parents indicated that they perceived these role-plays to be relevant and similar to their own experiences.

The themes discussed in our groups were reminiscent of those that emerged in prior group work with parents of gender diverse children and teens, including a strong focus on aspects of their children's gender journey and discussions related to parents' affective experiences. For example, Menvielle and Tuerk (2002) facilitated a group for parents of children under 12 years old, in which, similarly to our groups, parents discussed confusion around gender, expressing acceptance, supporting their children in coming out, concerns about peers and bullying, and grieving. In a more recent group consisting of parents of adolescents (Menvielle and Rodnan, 2011), several of the themes overlapped with our discussions including coming out, acceptance of gender, perceived losses, co-occurring mental health problems, and uncertainty about the future. Our groups varied in when and how deeply they focused on various themes, but all seemed to share a focus on these issues.

Of particular interest was whether the adapted program content adequately addressed relevant issues for caregivers of trans and gender non-conforming youth. Most parents reported that the group was helpful in enhancing their understanding of their child's gender identity; however, some parents still wanted a stronger focus on gender, and some expressed the need to discuss these issues earlier rather than later in the group. Interestingly, the group was designed to gradually address these themes, to help caregivers who began the group with ambivalence feel more comfortable prior to the discussion of gender related issues, because some parents in support groups may at times feel overwhelmed or judged (Hillier and Torg, 2019). In contrast, feedback indicated that parents expressed increasing satisfaction as the group progressed and there was a greater emphasis on specific issues related to gender. In future work it will be important to consider whether some flexibility in how quickly these issues are introduced is needed to adjust to diverse group composition.

With respect to outcomes, parent attendance and engagement in our groups was high and caregiver feedback supported the effectiveness of this intervention. Similarly, to other support groups (Menvielle and Tuerk, 2002; Rosenberg, 2002; Di Ceglie and Thümmel, 2006; Hillier and Torg, 2019), parents shared feeling isolated initially and relieved to be able to share their experiences with other parents who were encountering similar challenges. Although *Transforming Connections* is more experiential and less didactic in nature than the group described by Di Ceglie and Thümmel (2006) participants in both groups reported increased acceptance and understanding of their children. It remains to be determined whether the structured content in *Transforming Connections*, which is designed to support parents' capacity to reflect on their teen's challenges from an attachment perspective, to strengthen parental affect regulation, and promote understanding and empathy, is more helpful in promoting acceptance compared to parent support groups in the community, where the focus is largely on exchanging information and providing support for one other (Rosenberg, 2002; Lawlis et al., 2020). In fact, caregivers in our groups indicated that they liked both the structured and supportive aspects of the group. They reported feeling safe and supported, while also discussing strategies that allowed them to better support their teens.

Parents also endorsed rates of positive change in their relationships with their teens as a result of implementing these strategies, comparable to rates reported by parents who completed *Connect* (Moretti and Obsuth, 2009). Past research has shown that the improvements seen post *Connect* are long term and are evident even 2 years later (Högström et al., 2017). Thus, it is possible that this is the case for parents in this group, but it will be important to examine this further in future studies.

Of equal importance, parents expressed feeling more confident in parenting and expecting more positive changes in their relationship in the future. This increase in efficacy has been endorsed by parents who have completed the *Connect* group (Moretti and Obsuth, 2009) and may be related to the reported increased understanding of teens and how to respond in conflict, or the observed shifts in the relationships. Parents in our groups also discussed feeling reassured and relieved to understand many of the issues that their teens were struggling with were typical for their age. In future studies it will be important to examine this increase in confidence as a potential mechanism of change that mediates the positive outcomes (e.g., improved teen mental health) reported by both parents and teens post intervention. It may be that parents who are more confident in their parenting abilities are better able to cope with the challenges that they face.

Despite these promising preliminary results, there are a few limitations to consider. The sample size for this study was small and most parents identified as Caucasian. Recruitment was challenging as it appeared that parents initially found committing to ten sessions difficult. Menvielle and Tuerk (2002) highlighted that one obstacle to engaging parents is the stigma attached to gender non-conformity. Parents who participate in group interventions are likely to be more accepting of their teen's gender exploration (Malpas, 2011) due to a selection bias. In future studies, it would be helpful to explore this further and measure caregivers' level of acceptance of their teen's gender identity and gender journey pre- and post-participation in the group. It is also essential to continue to make an effort to recruit from a variety of settings in order to include a more diverse sample of participants.

Findings from the three pilot groups support the effectiveness of this intervention as a cost-effective method to deliver service to caregivers of trans and gender non-conforming youth. Results indicated that *Transforming Connections* was effective in reducing isolation experienced by caregivers of trans youth, promoting understanding and acceptance, and increasing parental efficacy by teaching parents to step back in their interactions with their teens. Feedback from these three pilot groups have informed further revisions of the program with the goal of co-creating a safe, helpful, gender-affirming, intervention. Future directions include training new leaders in order to build more capacity in the community and collecting data from parents and youth pre- and post-intervention.

## Data Availability Statement

The raw data supporting the conclusions of this article will be made available by the authors, without undue reservation.

## Ethics Statement

The studies involving human participants were reviewed and approved by Simon Fraser University REB. The patients/participants provided their written informed consent to participate in this study.

## Author Contributions

AD led the adaptation of the parenting program as part of her doctoral research and co-facilitated the three groups that were described in this paper, collected data, and analyzed the results. MM developed the Connect program, on which this adaptation was based. As AD's graduate research supervisor, MM supervised all aspects of the research project and provided clinical supervision and revisions to the doctoral dissertation, which formed this current manuscript. CB contributed to the adaptations of the parenting program and provided clinical supervision. CB also reviewed the manuscript, updated the relevant research, and contributed to the discussion. All authors contributed to the article and approved the submitted version.

## Conflict of Interest

The authors declare that the research was conducted in the absence of any commercial or financial relationships that could be construed as a potential conflict of interest.

## Publisher's Note

All claims expressed in this article are solely those of the authors and do not necessarily represent those of their affiliated organizations, or those of the publisher, the editors and the reviewers. Any product that may be evaluated in this article, or claim that may be made by its manufacturer, is not guaranteed or endorsed by the publisher.

## References

[B1] Adams N. Pearce R. Veale J. Radix A. Castro D. Sarkar A.. (2017). Guidance and ethical considerations for undertaking transgender health research and institutional review boards adjudicating this research. Transgender Health 2, 165–175. 10.1089/trgh.2017.0012 29098202 PMC5665092

[B2] Andrzejewski J. Pampati S. Steiner R. J. Boyce L. Johns M. M. (2021). Perspectives of transgender youth on parental support: qualitative findings from the resilience and transgender youth study. Health Educ. Behav. 48, 74–81. 10.1177/1090198120965504 33106050 PMC7962554

[B3] Bauer G. Devor A. Heinz M. Marshall Z. (2017). “Ethical guidelines for research involving trans people: launch of a new resource,” in Paper Presented at the Biennial Meeting of the Canadian Professional Association for Transgender Health (Vancouver, BC).

[B4] Brooks J. McCluskey S. Turley E. King N. (2015). The utility of template analysis in qualitative psychology research. Qual. Res. Psychol. 12, 202–222. 10.1080/14780887.2014.955224 27499705 PMC4960514

[B5] Clark T. C. Lucassen M. G. Bullen P. Denny S. J. Fleming T. M. Robinson E. M.. (2014). The health and well-being of transgender high school students: results from the New Zealand adolescent health suzrvey (Youth'12). J. Adolesc. Health 55, 93–99. 10.1016/j.jadohealth.2013.11.008 24438852

[B6] Di Ceglie D. Thümmel E. C. (2006). An experience of group work with parents of children and adolescents with gender identity disorder. Clin. Child Psychol. Psychiatry 11, 387–396. 10.1177/1359104506064983 17080775

[B7] Elliott R. Fischer C. T. Rennie D. L. (1999). Evolving guidelines for publication of qualitative research studies in psychology and related fields. Br. J. Clin. Psychol. 38, 215–229. 10.1348/014466599162782 10532145

[B8] Giannotta F. Ortega E. Stattin H. (2013). An attachment parenting intervention to prevent adolescents' problem behaviors: a pilot study in Italy. Child Youth Care Forum 42, 71–85. 10.1007/s10566-012-9189-3

[B9] Giovanardi G. Vitelli R. Maggiora Vergano C. Fortunato A. Chianura L. Lingiardi V.. (2018). Attachment patterns and complex trauma in a sample of adults diagnosed with gender dysphoria. Front. Psychol. 9:60. 10.3389/fpsyg.2018.00060 29449822 PMC5799708

[B10] Gray S. O. Carter A. S. Levitt H. (2012). A critical review of assumptions about gender variant children in psychological research. J. Gay Lesbian Mental Health 16, 4–30. 10.1080/19359705.2012.634719

[B11] Grossman A. H. D'Augelli A. R. (2007). Transgender youth and life-threatening behaviors. Suicide Life Threaten. Behav. 37, 527–537. 10.1521/suli.2007.37.5.527 17967119

[B12] Grossman A. H. Park J. Y. Frank J. A. Russell S. T. (2019). Parental responses to transgender and gender nonconforming youth: associations with parent support, parental abuse, and youths' psychological adjustment. J. Homosex. 68, 1260–1277. 10.1080/00918369.2019.1696103 31774377 PMC10676004

[B13] Hendricks M. L. Testa R. J. (2012). A conceptual framework for clinical work with transgender and gender nonconforming clients: an adaptation of the minority stress model. Prof. Psychol. 43, 460–467. 10.1037/a0029597

[B14] Hillier A. Torg E. (2019). Parent participation in a support group for families with transgender and gender-nonconforming children: “being in the company of others who do not question the reality of our experience.” Transgender Health 4, 168–175. 10.1089/trgh.2018.0018 31406916 PMC6689185

[B15] Högström J. Olofsson V. Özdemir M. Enebrink P. Stattin H. (2017). Two-year findings from a national effectiveness trial: effectiveness of behavioral and non-behavioral parenting programs. J. Abnorm. Child Psychol. 45, 527–542. 10.1007/s10802-016-0178-0 27334706

[B16] Holt V. Skagerberg E. Dunsford M. (2016). Young people with features of gender dysphoria: demographics and associated difficulties. Clin. Child Psychol. Psychiatry 21, 108–118. 10.1177/1359104514558431 25431051

[B17] Katz-Wise S. L. Ehrensaft D. Vetters R. Forcier M. Austin S. B. (2018). Family functioning and mental health of transgender and gender-nonconforming youth in the trans teen and family narratives project. J. Sex Res. 55, 582–590. 10.1080/00224499.2017.1415291 29336604 PMC7895334

[B18] Kozlowska K. Chudleigh C. McClure G. Maguire A. M. Ambler G. R. (2021). Attachment patterns in children and adolescents with gender dysphoria. Front. Psychol. 11:582688. 10.3389/fpsyg.2020.582688 33510668 PMC7835132

[B19] Lawlis S. M. Butler P. Middleman A. (2020). Evaluating transgender youth and parent interest and preferences regarding support groups. Glob. Pediatr. Health 7:2333794X20954680. 10.1177/2333794X20954680 32964072 PMC7488600

[B20] Malpas J. (2011). Between pink and blue: a multi-dimensional family approach to gender nonconforming children and their families. Fam. Process 50, 453–470. 10.1111/j.1545-5300.2011.01371.x 22145719

[B21] Menvielle E. Hill D. B. (2010). An affirmative intervention for families with gender-variant children: a process evaluation. J. Gay Lesbian Mental Health 15, 94–123. 10.1080/19359705.2011.530576 20063232

[B22] Menvielle E. J. Rodnan L. A. (2011). A therapeutic group for parents of transgender adolescents. Child Adolesc. Psychiatr. Clin. N. Am. 20, 733–743. 10.1016/j.chc.2011.08.002 22051009

[B23] Menvielle E. J. Tuerk C. (2002). A support group for parents of gender-nonconforming boys. J. Am. Acad. Child Adolesc. Psychiatry 41, 1010–1013. 10.1097/00004583-200208000-00021 12162618

[B24] Moretti M. M. (2019). “Adolescence, attachment and intervention: promoting resilience during risky transitions,” in Keynote presented at the 9th International Attachment Conference (IAC), July 18th-20th (Vancouver, BC).

[B25] Moretti M. M. (2020). Connect: An Attachment Based and Trauma Informed Program for Parents and Caregivers, 3rd Edn. Burnaby, BC: Simon Fraser University.

[B26] Moretti M. M. Obsuth I. (2009). Effectiveness of an attachment-focused manualized Intervention for parents of teens at risk for aggressive behaviour: the connect program. J. Adolesc. 32, 1347–1357. 10.1016/j.adolescence.2009.07.013 19766302

[B27] Moretti M. M. Obsuth I. Craig S. G. Bartolo T. (2015). An attachment-based Intervention for parents of adolescents at risk: mechanisms of change. Attach. Hum. Dev. 17, 119–135. 10.1080/14616734.2015.1006383 25782460

[B28] Moretti M. M. Obsuth I. Mayseless O. Scharf M. (2012). Shifting internal parent-child representations among caregivers of teen with serious behaviour problems: an attachment based approach. J. Child Adolesc. Trauma 5, 191–204. 10.1080/19361521.2012.697104

[B29] Moretti M. M. Pasalich D. S. O'Donnell K. A. (2017). “Connect: an attachment-based program for parents of teens,” in Handbook of Attachment-Based Interventions, eds Steele H. Steele M. (New York, NY: Guilford Press), 375–400.

[B30] Osman F. Flacking R. Schon U.-K. Klingberg-Allvin M. (2017). A support program for Somali-born parent on children's behavioural problems. Pediatrics 139, 1–9. 10.1542/peds.2016-2764 28235795

[B31] Ozturk Y. Moretti M. Barone L. (2019). Addressing parental stress and adolescents? behavioral problems through an attachment-based program: An intervention study. Int. J. Psychol. Psychol. Ther. 19, 89–100.

[B32] QSR International Pty Ltd (2015). NVivo qualitative Data Analysis Software - Version 11. Doncaster, VIC: Author.

[B33] Reisner S. L. Greytak E. A. Parsons J. T. Ybarra M. L. (2014). Gender minority social stress in adolescence: disparities in adolescent bullying and substance use by gender identity. J. Sex Res. 52, 243–256. 10.1080/00224499.2014.886321 24742006 PMC4201643

[B34] Reisner S. L. Vetters R. Leclerc M. Zaslow S. Wolfrum S. Shumer D.. (2015). Mental health of transgender youth in care at an adolescent urban community health center: a matched retrospective cohort study. J. Adolesc. Health 56, 274–279. 10.1016/j.jadohealth.2014.10.264 25577670 PMC4339405

[B35] Riley E. A. Sitharthan G. Clemson L. Diamond M. (2013). Recognizing the needs of gender-variant children and their parents. Sex Educ. 13, 644–659. 10.1080/14681811.2013.796287 23356537

[B36] Rosenberg M. (2002). Children with gender identity issues and their parents in individual and group treatment. J. Am. Acad. Child Adolesc. Psychiatry 41, 619–621. 10.1097/00004583-200205000-00020 12014795

[B37] Ryan C. Russell S. T. Huebner D. Diaz R. Sanchez J. (2010). Family acceptance in adolescence and the health of LGBT young adults. J. Child Adolesc. Psychiatr. Nurs. 23, 205–213. 10.1111/j.1744-6171.2010.00246.x 21073595

[B38] Schumm W. R. Crawford D. W. (2020). Is research on transgender children what it seems? Comments on recent research on transgender children with high levels of parental support. The Linacre Q. 87, 9–24. 10.1177/0024363919884799 32431444 PMC7016423

[B39] Simon K. A. Farr R. H. (2020). Development of the conceptual future parent grief (CFPG) scale for LGBTQ+ people. J. Fam. Psychol. 35, 299–310. 10.1037/fam0000790 32700927

[B40] Simons L. Schrager S. M. Clark L. F. Belzer M. Olson J. (2013). Parental support and mental health among transgender adolescents. J. Adolesc. Health 53, 791–793. 10.1016/j.jadohealth.2013.07.019 24012067 PMC3838484

[B41] Stattin H. Enebrink P. Özdemir M. Giannotta F. (2015). A national evaluation of parenting programs in Sweden: The short-term effects using an RCT effectiveness design. J. Consult. Clin. Psychol. 83(6), 1069–1084. 26009784 10.1037/a0039328

[B42] Taylor A. B. Chan A. Hall S. L. Saewyc E. M. the Canadian Trans Non-binary Youth Health Survey Research Group (2020). Being Safe, Being Me 2019: Results of the Canadian Trans and Non-binary Youth Health Survey. Vancouver, BC: Stigma and Resilience Among Vulnerable Youth Centre, University of British Columbia.

[B43] Travers R. Bauer G. Pyne J. Bradley K. Gale L. Papadimitriou M. (2012). Impacts of Strong Parental Support for Trans Youth: A Report Prepared for Children's Aid Society of Toronto and Delisle Youth Services. Available online at: https://transpulseproject.ca/wp-content/uploads/2012/10/Impacts-of-Strong-Parental-Support-for-Trans-Youth-vFINAL.pdf

[B44] Veale J. Saewyc E. Frohard-Dourlent H. Dobson S. Clark B. the Canadian Trans Youth Health Survey Research Group. (2015). Being Safe, Being Me: Results of the Canadian Trans Youth Health Survey. Vancouver BC: Stigma and Resilience Among Vulnerable Youth Centre, School of Nursing, University of British Columbia.

[B45] Veale J. F. Watson R. J. Peter T. Saewyc E. M. (2017). Mental health disparities among Canadian transgender youth. J. Adolesc. Health 60, 44– 10.1016/j.jadohealth.2016.09.014 28007056 PMC5630273

